# The plastid genome of twenty-two species from *Ferula*, *Talassia*, and *Soranthus*: comparative analysis, phylogenetic implications, and adaptive evolution

**DOI:** 10.1186/s12870-022-04027-4

**Published:** 2023-01-05

**Authors:** Huan-Huan Qin, Jing Cai, Chang-Kun Liu, Ren-Xiu Zhou, Megan Price, Song-Dong Zhou, Xing-Jin He

**Affiliations:** 1 Key Laboratory of Bio-Resources and Eco-Environment of Ministry of Education, College of Life Sciences, Sichuan University, Chengdu, 610065 China; 2 Key Laboratory of Conservation Biology On Endangered Wildlife, College of Life Sciences, Sichuan University, Chengdu, 610065 China

**Keywords:** Apiaceae, *Ferula*, *Talassia* and *Soranthus*, Plastome evolution, DNA barcoding, Phylogenetic relationships

## Abstract

**Background:**

The *Ferula* genus encompasses 180–185 species and is one of the largest genera in Apiaceae, with many of *Ferula* species possessing important medical value. The previous studies provided more information for *Ferula*, but its infrageneric relationships are still confusing. In addition, its genetic basis of its adaptive evolution remains poorly understood. Plastid genomes with more variable sites have the potential to reconstruct robust phylogeny in plants and investigate the adaptive evolution of plants. Although chloroplast genomes have been reported within the *Ferula* genus, few studies have been conducted using chloroplast genomes, especially for endemic species in China.

**Results:**

Comprehensively comparative analyses of 22 newly sequenced and assembled plastomes indicated that these plastomes had highly conserved genome structure, gene number, codon usage, and repeats type and distribution, but varied in plastomes size, GC content, and the SC/IR boundaries. Thirteen mutation hotspot regions were detected and they would serve as the promising DNA barcodes candidates for species identification in *Ferula* and related genera. Phylogenomic analyses with high supports and resolutions showed that *Talassia transiliensis* and *Soranthus meyeri* were nested in the *Ferula* genus, and thus they should be transferred into the *Ferula* genus. Our phylogenies also indicated the monophyly of subgenera *Sinoferula* and subgenera *Narthex* in *Ferula* genus. Twelve genes with significant posterior probabilities for codon sites were identified in the positively selective analysis, and their function may relate to the photosystem II, ATP subunit, and NADH dehydrogenase. Most of them might play an important role to help *Ferula* species adapt to high-temperatures, strong-light, and drought habitats.

**Conclusion:**

Plastome data is powerful and efficient to improve the support and resolution of the complicated *Ferula* phylogeny. Twelve genes with significant posterior probabilities for codon sites were helpful for *Ferula* to adapt to the harsh environment. Overall, our study supplies a new perspective for comprehending the phylogeny and evolution of *Ferula*.

**Supplementary Information:**

The online version contains supplementary material available at 10.1186/s12870-022-04027-4.

## Background

*Ferula* L. is one of the genera of Apiaceae [1], which was once classified in the tribe Peucedaneae [2, 3], but now in the tribe Scandiceae [4–6]. This genus, encompassing about 180–185 species all over the world [7], distributes in the Mediterranean region, Siberia, Central Asia, and northern Africa [3, 8, 9], and grows mostly in mountainous regions and desert clay soils [8, 10]. The *Ferula* genus has been chiefly recognized by the prominent taproots, stout stems, finely divided leaves with large inflated sheaths, and strongly dorsally compressed mericarps with filamentary or prominent dorsal ribs, narrowly or broadly winged marginal ribs and the plane or slightly concave commissural face [1, 6]. However, due to the great variations in the leaf, inflorescences, and mericarps anatomy, distinguishing this genus from nearby genera was extremely difficult. Hence, the taxonomic delimitation of *Ferula* has long been contentious. Pimenov [11, 12] suggested that *Talassia* and *Soranthus* should be transferred into *Ferula* according to the anatomical characteristics of the fruit which was the presence of a sclerotic cell layer in the mesocarp of fruits. Pimenov [13], according to the type specimens and morphological features, summarized the nomenclatural combinations of *Ferula* in China and merged the *S. meyeri* and *T. transiliensis* into the *Ferula*. However, Qin and Shen [14] believed that *Ferula* L., *Soranthus* Ledeb., and *Talassia* Korov. should exist as separate genera in Apiaceae, based on the comparison of the external morphology, fruit anatomy, and pollen characteristics of the plants. In Flora of China [1] and The Flora of Reipublicae Popularis Sinica [15], *Soranthus* and *Talassia* were also separated from the *Ferula*. Therefore, the generic limits between the *Ferula* and its nearby genera based solely on morphological characteristics was challenging.

Before, scholars have recently used molecular data to study the taxonomy and phylogeny of *Ferula* and its relative nearby genera. Dowine et al. [16] summarizing the previous study results, proposed that *Talassia* and *Soranthus* were closely related to *Ferula* but more research is needed to resolve the relationship. Kurzyna-Młynik et al. [6] and Panahi et al. [17, 18] have placed the *T. transiliensis* and *S. meyeri* into *Ferula* according to the phylogenetic trees using the nuclear ribosomal DNA internal sequence data (ITS) and three plastid non-coding regions. But the support and resolution in these phylogenetic trees were weak and low, and thus the phylogenetic position of *T. transiliensis* and *S. meyeri* within *Ferula* genus was unresolved. So additional markers are needed to obtain a robust phylogeny.

The infrageneric taxonomic system of *Ferula* has been complicated. Based on habit and vegetative characteristics, Korovin [19] established the six subgenera and eight sections of this genus. And this division was adopted in The Flora of Reipublicae Popularis Sinica [15] where the *Ferula* species grown in China were divided into four sections and four subgenera. However, Safina and Pimenov [20–22] contested the infrageneric division provided by Korovin and proposed 12 new sections of *Ferula* genus based on mericarps morphology and anatomy. Nevertheless, subsequent molecular study did not agree with those infrageneric taxonomies, and inferred a new infrageneric classification of *Ferula*. Panahi et al. [17] used nrITS and three plastid non-coding regions to propose a new classification system for *Ferula* of four subgenera and ten sections, where the species growing in China were divided into two subgenera.

Besides, many species of *Ferula* have medical value and are extensively used in traditional medicine in folk and pharmacy. For example, *F. sinkiangensis* K. M. Shen and *F. fukanensis* K. M. Shen are used as vital traditional medicines to eliminate stagnation, resolve symptoms, disperse lumps, and kill worms [23, 24]. Other species, such as *F. lehmannii* Boiss., *F. songarica* Pall. ex Spreng., *F. olivacea* (Diels) H. Wolff ex Hand. -Mazz., and *F. feruloides* (Steud.) Korovin, also have significant pharmaceutical effects [25]. However, due to the high market value and morphological similarities, the other *Ferula* species are usually used as substitutes for *F. sinkiangensis* and *F. fukanensis*. Consequently, it is indispensable to develop more DNA barcodes for species authentication to ensure medicinal quality.

The plastid is an essential organelle for green plants, which is responsible for photosynthesis and offers the basic energy for plants [26]. The plastid genome (plastome) is uniparentally inherited, lacks recombination, has low nucleotide substitution rates, and contains abundant variable sites. Therefore, the plastome is a useful tool to improve the certainty of phylogenetic trees [27, 28]. The plastome generally is 115 to 165 kb in length, containing a large single-copy region (LSC), two separately inverted repeat regions (IRs), and one small single-copy region (SSC), and encodes about 110–130 unique genes [29–31]. Comparative analysis of plastomes reveals the variation in its structural combination and gene arrangement, which is helpful to further identify the mutational hotspots for species authentication [32, 33]. Consequently, with the processing development of next-generation sequencing and multiple bioinformatics technologies, plastomes have been broadly and successfully applied to the development of DNA barcodes and analysis of phylogenetics [34, 35]. In addition, the plastomes are used to investigate the adaptive evolution of plants. Adaptive evolution implies that the adaptability of species is enhanced during the evolutionary processes, driven by the natural selective pressure applied to the genetic variation through gene flow, recombination, and mutations [36] and causes biodiversity in each aspect of biological organization [37]. Understanding the adaptive evolution of organisms could contribute to elucidating the latent mechanism of adapting to the local environment and providing guidance for future protection [38, 39]. For example, *accD*, *rpoA*, and *rpoC2* genes were positively selected in the *Rehmannia* species, which helped species to grow in divergent light intensity habits [40]. Furthermore, *psbH*, *psbM*, and *rbcL* genes may work in the growth of all Dipterocarpoideae species to adapt to a strongly illuminated environment [41]. As for *Ferula* genus, limited chloroplast genome data has been reported [42], and few studies, especially for focusing on endemic species in China, have been conducted using chloroplast genomes.

Here, with newly sequenced 22 plastomes of *Ferula*, *Talassia*, and *Soranthus* species, we analyzed 42 plastomes from the Apiaceae subfamily and aimed to (1) evaluate the infrageneric classification system of *Ferula*; (2) exploit promising candidate DNA markers of this genus; and (3) investigate the adaptive evolution of this genus based on plastome data. In brief, our study will enhance knowledge of the phylogeny and adaptive evolution of *Ferula*.

## Results

### Features of the plastome

**Fig. 1:**
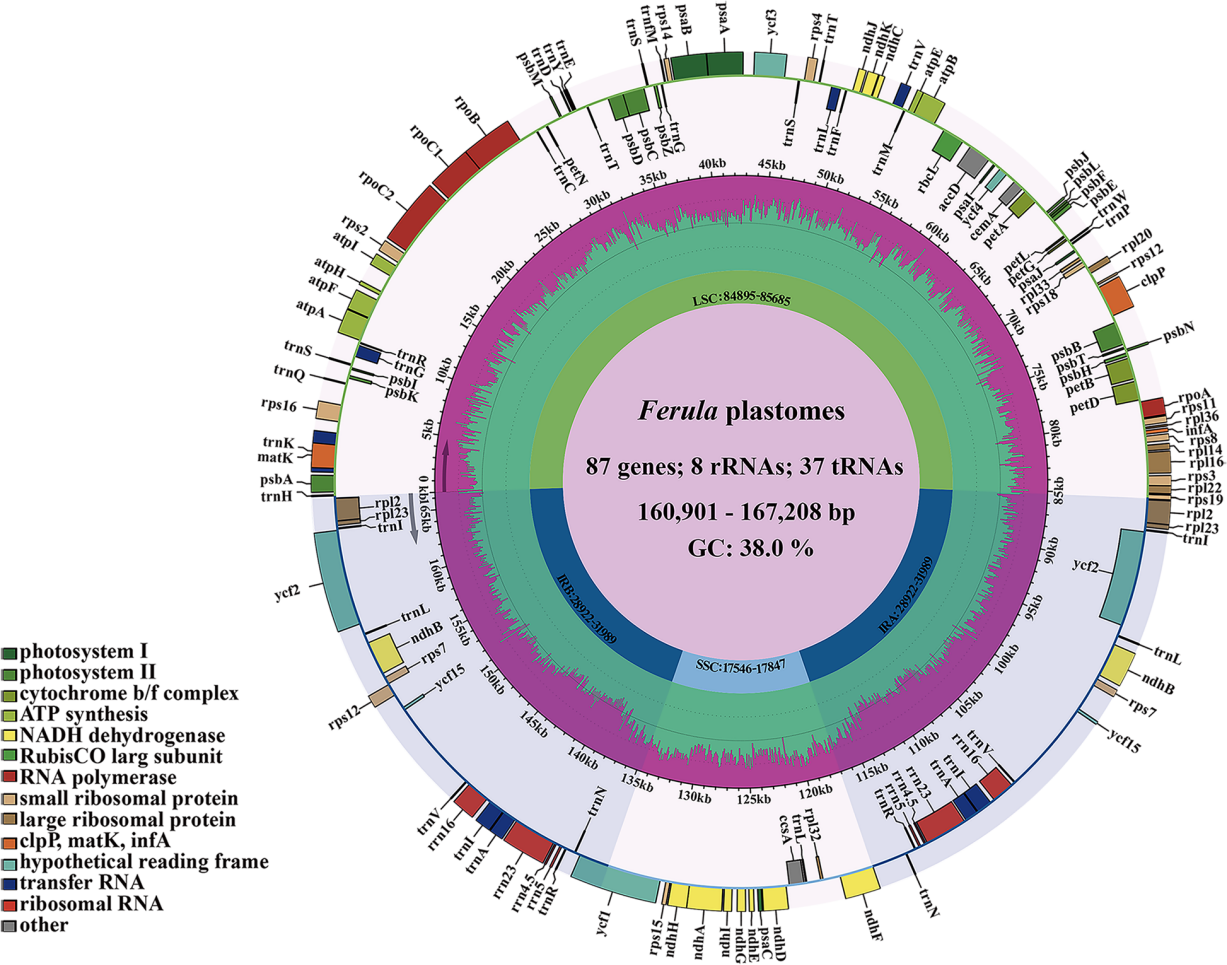
Plastome maps of twenty-two species. Genes shown outside of outward layer circle are transcribed clockwise, while those insides are transcribed counterclockwise. The genes belonging to different functional groups are color-coded. The green area of the inner circle denotes the GC content of plastome

The plastomes of 22 species ranged from 160,901 bp (*F. conocaula*) to 167,208 bp (*F. olivacea*) in length (Table 1). All plastomes possessed the typical quadripartite structure with two copies of IR regions (28,922–31,989 bp) separated by the LSC region (84,904 -85,895 bp) and SSC region (17,546–17,846 bp). The total GC content was between 37.6 and 38.0%, and the IR regions were the highest (42.8–43.1%) compared to the LSC (35.5–35.7%) and SSC regions (30.6–31.1%). The rRNA genes had the highest GC content, greater than the tRNA genes and protein-coding genes. Each of these 22 plastomes contained 133 genes, consisting of 87 protein-coding genes, 37 tRNA genes, and eight rRNA genes (Fig. 1, Table 1). Of these genes, 14 genes contained one intron, and four genes contained two introns (Fig. 1, Table S1).

**Table 1:** The plastome features of 22 species

Taxa	Total length(bp)	LSC (bp)	SSC (bp)	IR (bp)	Total GC(%)	LSC (%)	SSC (%)	IR (%)	Protein-coding region(%)	rRNA(%)	tRNA (%)	Total genes number	Protein-coding gene	rRNA genes	tRNA gene
*F. sinkiangensis*	166,482	85,190	17,568	31,862	38.0	35.7	31.1	43.0	38.1	55.3	53.2	133	87	8	37
*F. leiophylla*	166,492	85,215	17,571	31,853	38.0	35.7	31.1	43.0	38.1	55.3	53.2	133	87	8	37
*F. teterrica*	166,489	85,193	17,584	31,856	38.0	35.7	31.1	43.0	38.1	55.3	53.3	133	87	8	37
*F. akitschkensis*	166,404	85,271	17,587	31,773	38.0	35.7	31.0	43.0	38.1	55.3	53.2	133	87	8	37
*F. feruloides*	166,436	85,269	17,587	31,790	38.0	35.7	31.1	43.0	38.1	55.3	53.1	133	87	8	37
*F. songarica*	166,437	85,274	17,561	31,801	38.0	35.7	31.1	43.0	38.1	55.3	53.1	133	87	8	37
*F. lehmannii*	166,542	85,303	17,629	31,805	38.0	35.7	31.0	43.0	38.1	55.3	53.2	133	87	8	37
*F. gracilis*	166,522	85,203	17,587	31,866	38.0	35.7	31.0	43.0	38.1	55.3	53.2	133	87	8	37
*F. canescens*	165,135	84,895	17,600	31,320	37.9	35.7	31.1	42.9	38.1	55.3	53.1	133	87	8	37
*F. caspica*	161,878	85,203	17,569	29,553	37.9	35.6	31.1	43.1	38.1	55.3	53.2	133	87	8	37
*F. bungeana*	166,526	85,326	17,624	31,788	37.9	35.6	31.0	43.0	38.0	55.3	53.2	133	87	8	37
*F. licentiana*	166,642	85,343	17,625	31,837	37.9	35.6	31.0	43.0	38.0	55.3	53.3	133	87	8	37
*F. dissecta*	166,461	85,352	17,561	31,774	38.0	35.6	31.1	43.0	38.1	55.3	53.2	133	87	8	37
*F. hexiensis*	166,378	84,904	17,568	31,953	37.9	35.6	31.0	42.9	38.0	55.3	53.1	133	87	8	37
*F. conocaula*	160,901	85,179	17,546	29,088	37.9	35.7	31.1	43.1	38.1	55.3	53.1	133	87	8	37
*T. transiliensis*	166,520	85,293	17,585	31,821	38.0	35.7	31.0	43.0	38.1	55.3	53.1	133	87	8	37
*F. syreitschikowii*	166,590	85,300	17,640	31,825	38.0	35.6	31.0	43.0	38.0	55.3	53.0	133	87	8	37
*S. meyeri*	166,651	85,352	17,631	31,834	37.9	35.6	31.0	43.0	38.1	55.3	53.1	133	87	8	37
*F. kirialovii*	166,068	85,220	17,674	31,587	38.0	35.7	31.0	43.0	38.1	55.3	53.2	133	87	8	37
*F. olivacea*	167,208	85,520	17,710	31,989	37.8	35.5	30.7	42.9	38.0	55.3	52.9	133	87	8	37
*F. paeoniifolia*	167,112	85,551	17,661	31,950	37.8	35.5	30.8	42.9	38.0	55.3	53.2	133	87	8	37
*F. kingdon-wardii*	161,376	85,685	17,847	28,922	37.6	35.5	30.6	42.8	38.0	55.3	53.0	133	87	8	37

### Repeat sequences analysis and codon usage

**Fig. 2:**
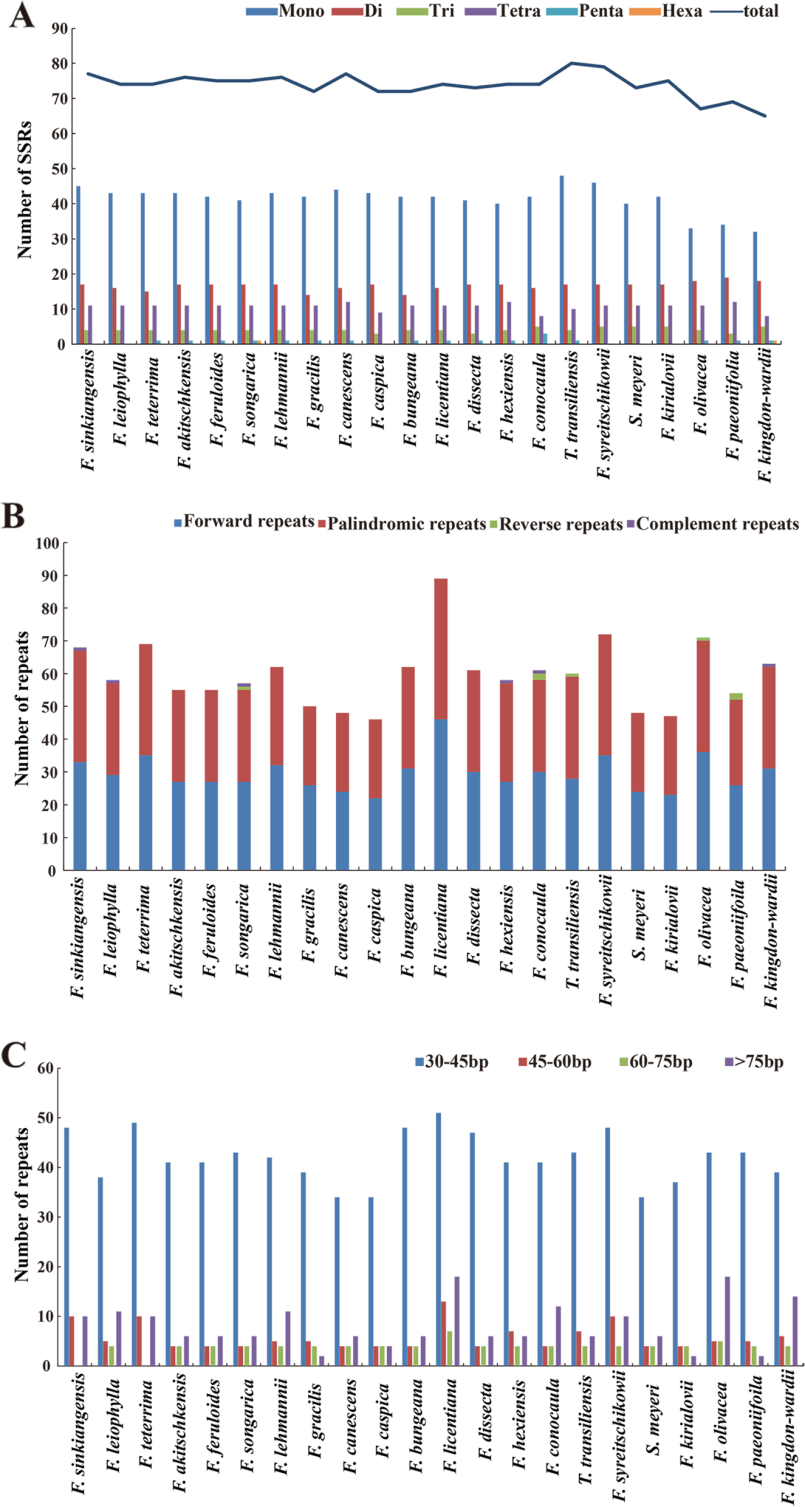
Analysis of simple sequence repeats (SSRs) and repeat sequences in 22 species plastomes. **A** Total numbers of various repeat types. **B** Total Numbers of different repeat types. **C** Number of repeats divided by length

The total number of SSRs ranged from 65 (*F. kingdon-wardii*) to 80 (*T. transiliensis*) within the 22 plastomes (Fig. 2A). The most abundant were mononucleotide repeats (32–48), followed by dinucleotides (14–19), tetranucleotides (8–12), trinucleotides (3–5), and pentanucleotides (0–3). Only *F. songarica* and *F. kingdon-wardii* had one hexanucleotide (Fig. 2A). (T)10 was found in the intergenic region between *atpH* and *atpI* in only *F. olivacea*, *F. paeoniifolia*, and *F. kingdon-wardii*. We also found (ATTA)3 was distributed in the coding region of *rps2* in *F. olivacea* and *F. paeoniifolia*. (G)10 or (G)11 was allocated at the intergenic region (*psbZ*/*trn*G) in *F. olivacea* and *F. paeoniifolia*, while (AAAT)3 was only found in the intergenic region (*trn*S /*psbZ*) in *F. kirialovii*, and (A)15 was observed in the *ndhF* gene only in *T. transiliensis*, and so on (Table S2). SSRs were distributed largely in the LSC region, less in the SSC and IR regions. Moreover, the analysis of SSRs locations uncovered that the majority of SSRs were distributed in the non-coding regions that contained the intron and the intergenic regions (Table S2). In addition, the forward, palindromic, complementary, and reverse repeats were detected in the 22 species, and the total number of repeats was 1,314. The forward repeats were the most abundant (649), while the complement repeats were the least (6) (Fig. 2B, Table S3). Among the 22 species, the *F. licentiana* had the most repeats (89), while *F. caspica* possessed the least repeats (46). In addition, we divided the repeats into four types according to length: 30–45 bp, 45–60 bp, 60–75 bp, and > 70 bp, and most of the repeats (70.32%) were 30–45 bp long (Fig. 2C).

**Fig. 3:**
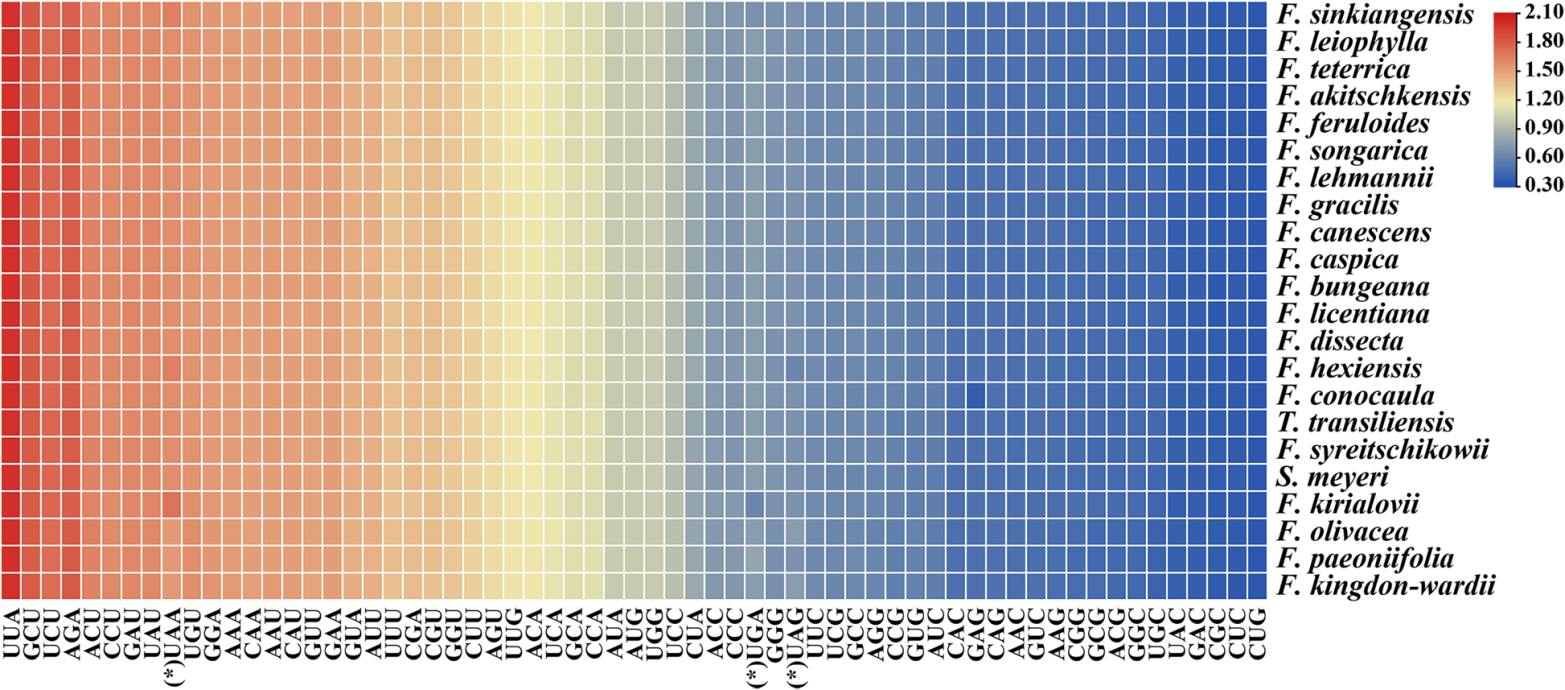
The RSCU values of all concatenated protein-coding genes for 22 species plastomes. Color key: the red values mean higher RSCU values and the blue values mean lower RSCU values. (*) to mark the terminator codons

We extracted and connected 53 protein-coding genes in each species to characterize the codon usage of 22 plastomes (Fig. 3, Table S4). These protein sequences encoded 21,087–21,185 codons (Table S4). Among them, Leu, Ser, and Arg were encoded by six codons indicating the highest preference, and Leu was most abundant (2,092–2,234), while the Cys was least (217–221) in all plastomes (Table S4). Additionally, relative synonymous codon usage (RSCU) values of all codons ranged from 0.31 to 2.00 in all species, and the RSCU values of about 30 codons were greater than 1(Fig. 3).

### Comparison of plastomes

**Fig. 4:**
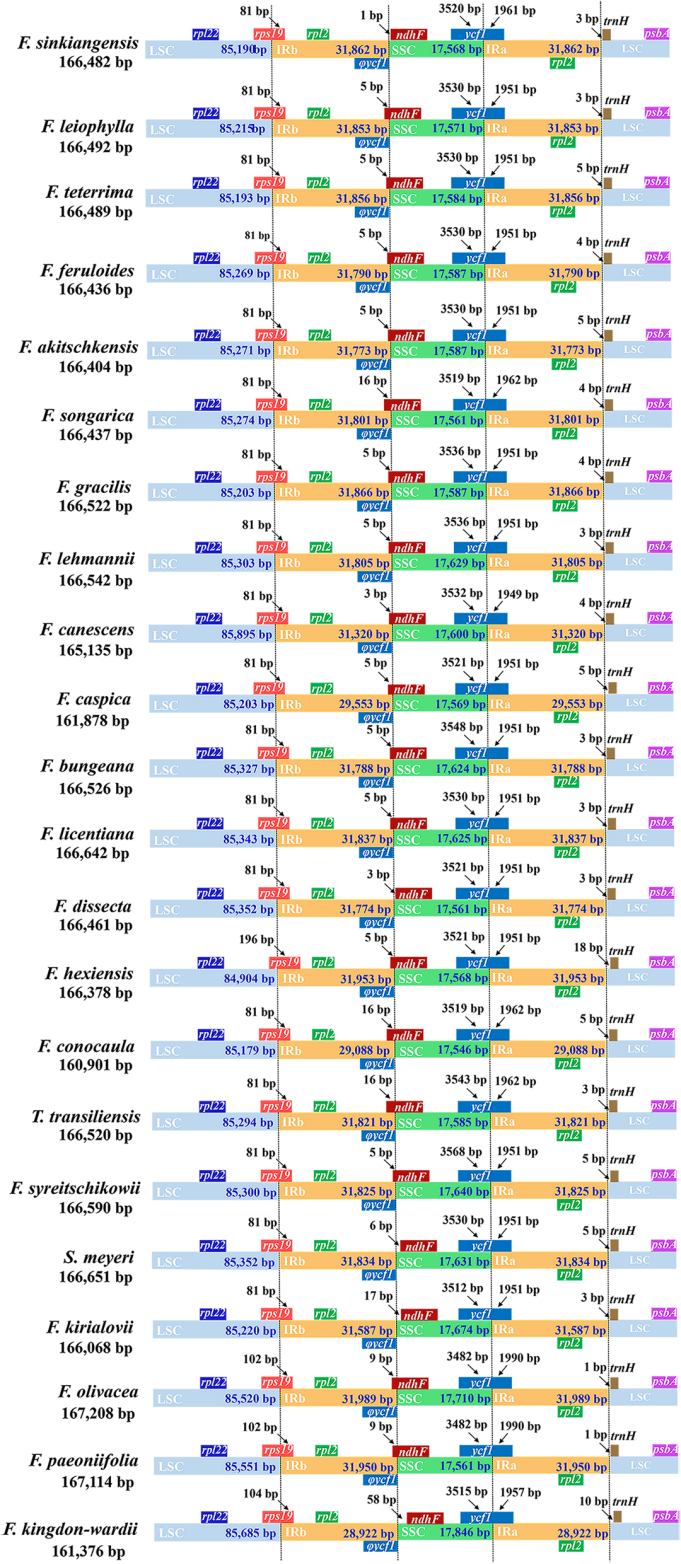
Comparison of the border of the LSC, SSC, and IR regions among twenty-two species plastomes

The borders of LSC/IRb, IRb/SSC, SSC/IRa, and IRa/LSC among the 22 plastomes were relatively conserved and similar (Fig. 4). The LSC/IRb borders were fell into *rps19*; IRb/SSC borders were fell into the *ndhF* gene, but located between the *ycf1* and *ndhF* genes in *F. sinkiangensis*, *F. dissecta*, *S. meyeri*, *F. kirialovii*, and *F. kingdon-wardii*; the SSC/IRa borders were fell into ψ*ycf1* gene and the IRa/LSC borders were located between the *rpl2* gene and *trn*H gene.

**Fig. 5:**
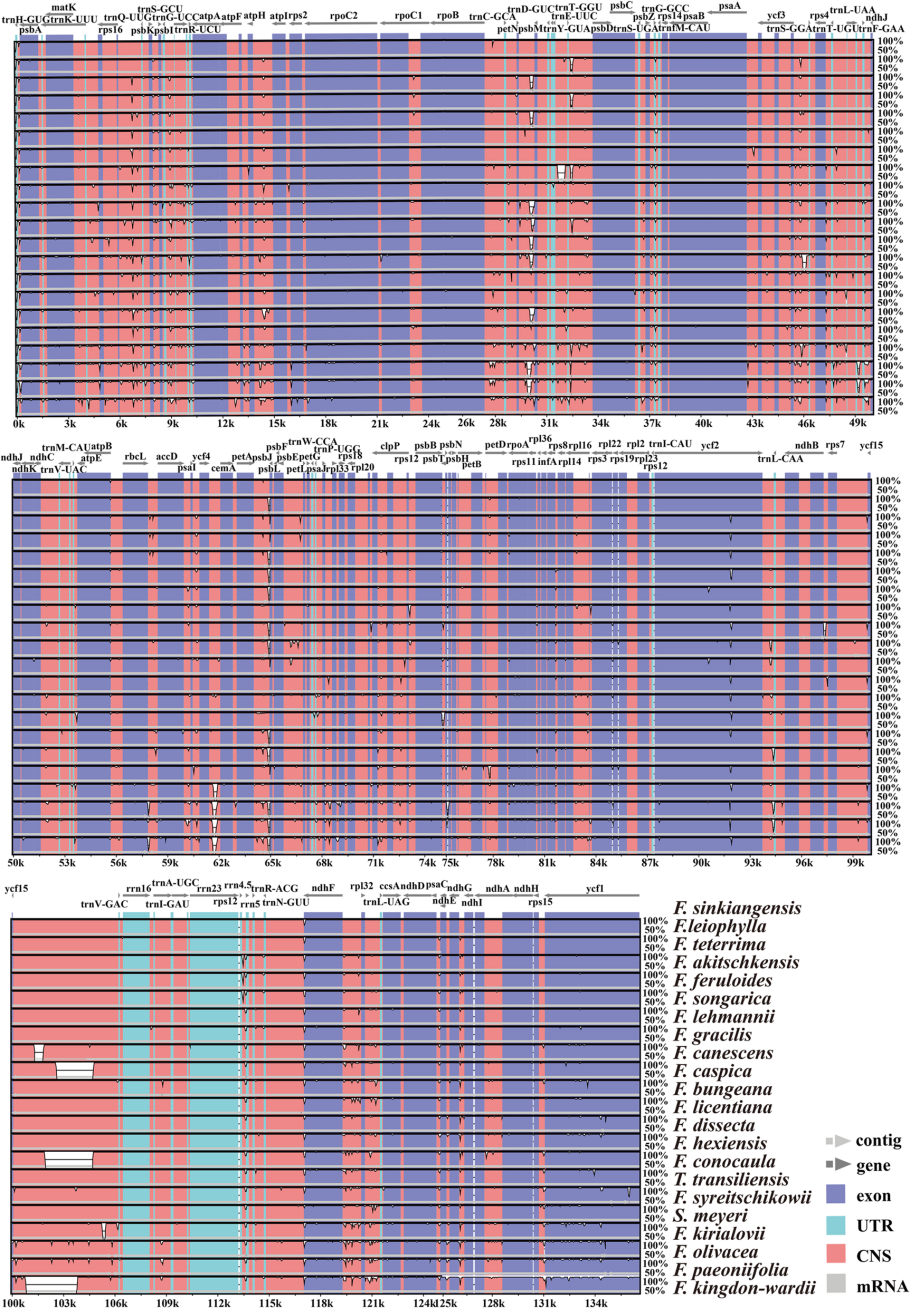
Sequence identity plots of the 22 species plastomes using *F. sinkiangensis* as a reference

**Fig. 6:**
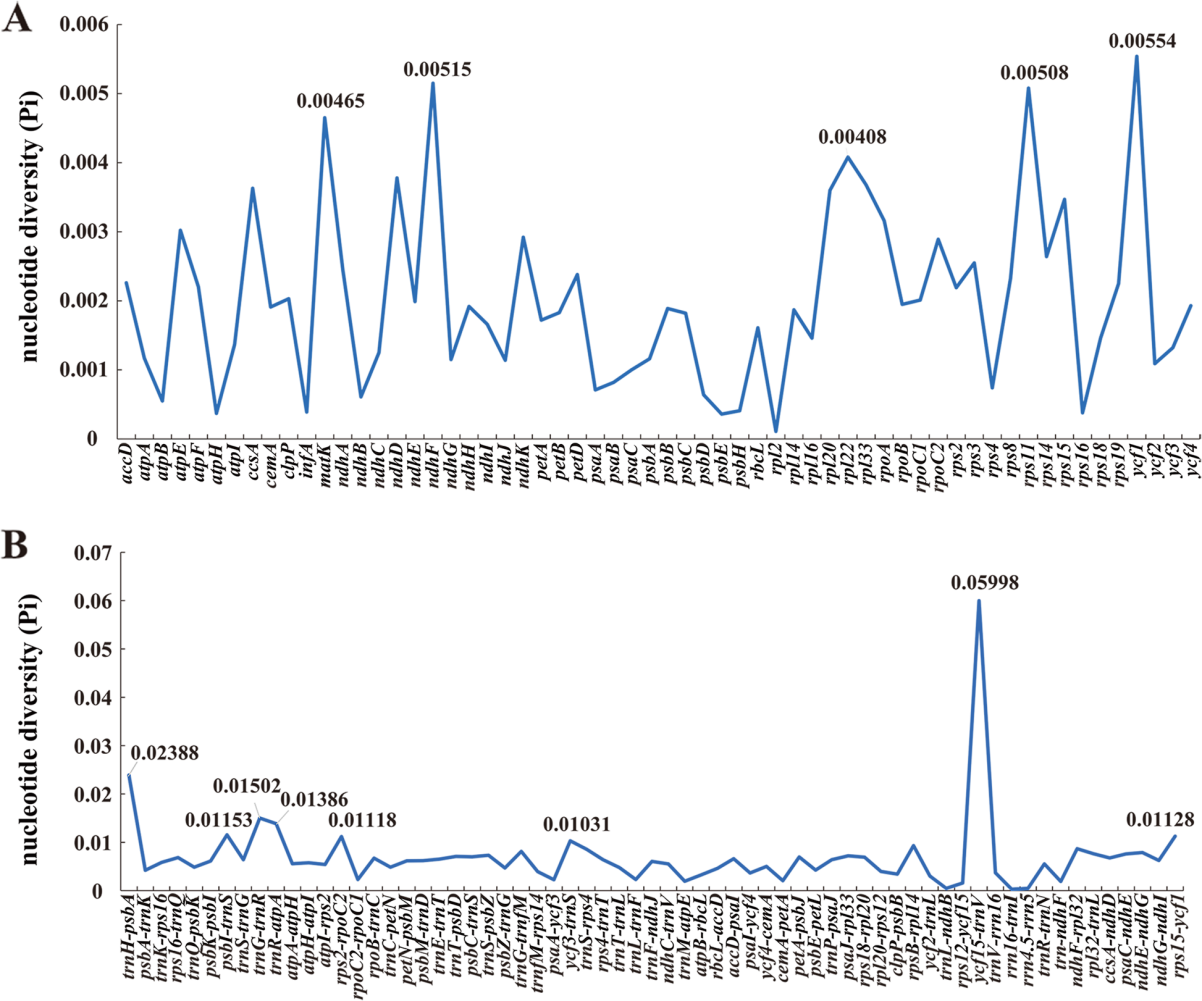
Comparative analysis of the nucleotide diversity (Pi) values among the twenty-two species plastomes: **A** coding regions; **B** non-coding regions

Using the mVISTA program, we found that the plastomes of the 22 taxa were highly conserved, and the IR regions and coding regions were more conserved than the SC regions and non-coding regions (Fig. 5). Nevertheless, 13 hotspot regions were detected, including five coding regions (*ycf1*, *ndhF*, *rps11*, *matK*, and *rpl22*) that possessed Pi > 0.004 and eight non-coding regions (*ycf1*5/*trn*V, *trn*H /*psbA*, *trn*G/*trn*R, *trn*R /*atpA*, *psbI*/*trn*S, *rps15*/*ycf1*, *rps2*/*rpoC2*, and *ycf3*/*trn*S) that had Pi > 0.010 (Fig. 6). In total, these regions could be used for DNA barcode studies in the future.

### Phylogenetic relationships

**Fig. 7:**
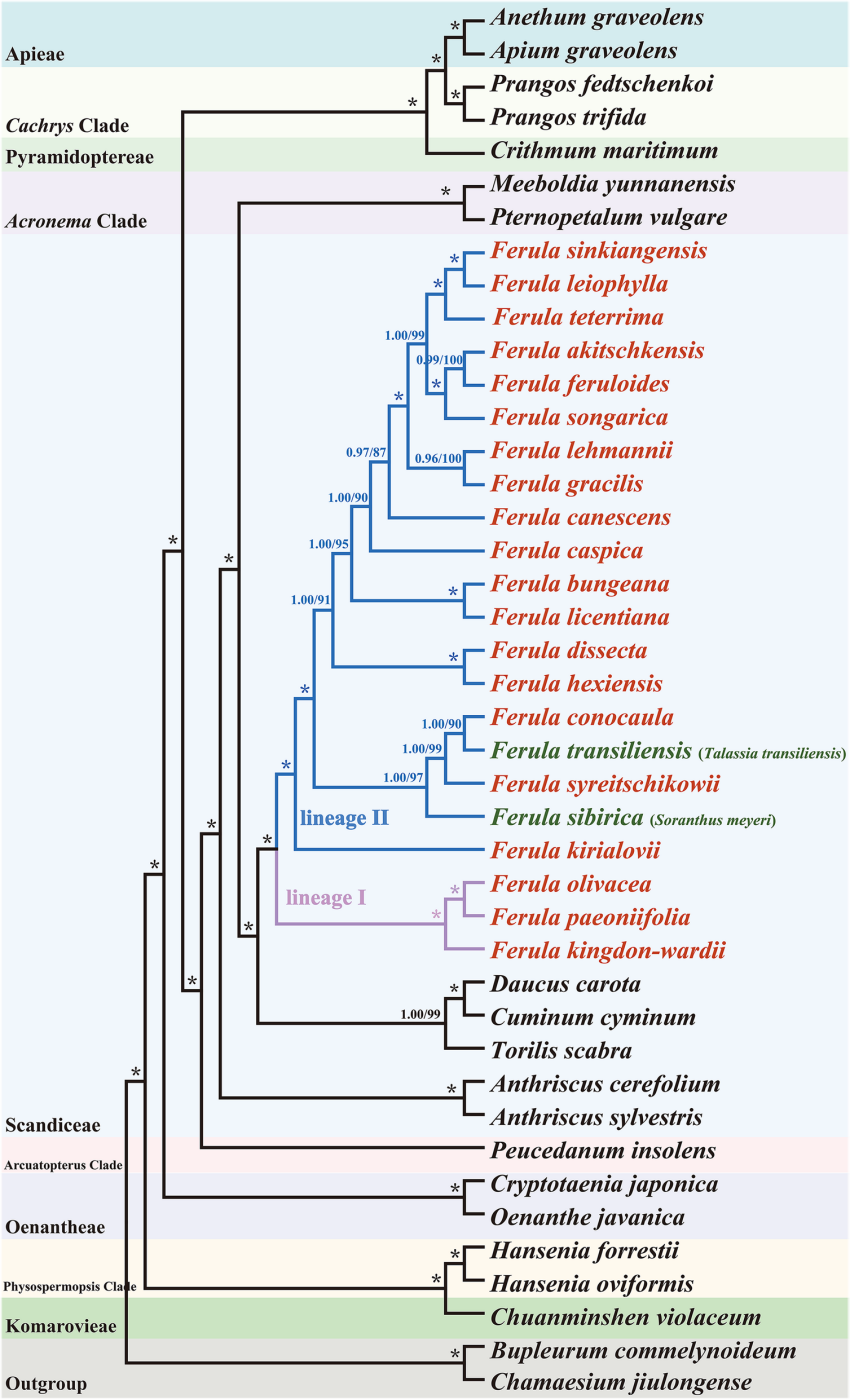
Phylogenetic tree reconstruction of 42 taxa inferred from Maximum likelihood (ML) and Bayesian inference (BI) analyses based on the single-copy CDs. Numbers indicate Bayesian posterior probabilities (PP) and maximum likelihood bootstrap values (BS), and (*) indicates maximum support in both two analyses

We reconstructed the phylogenetic trees based on single-copy CDs of 42 species plastomes and 62 ITS sequence data (Table S5). The plastomes tree and ITS tree showed incongruent topologies, but the trees indicated that *T. transiliensis* and *S. meyeri* nested within *Ferula* species (Fig. 7; Fig. S1). In the plastome tree, the ML (maximum likelihood) and BI (Bayesian inference) analyses resulted in identical trees, and both analyses strongly showed that the *T. transiliensis*, *S. meyeri*, and *Ferula* members formed a robustly supported clade (BS > 85%, PP ≥ 0.95). *Soranthus meyeri* was clustered with the branch formed by *F. syreitschikowii*, *T. transiliensis*, and *F. conocaula* where *F. syreitschikowii* was the sister to a subclade consisting of the *T. transiliensis* and *F. conocaula* (BS = 90, PP = 1.00), and then they nested within the *Ferula* members (BS ≥ 90, PP ≥ 0.99). Furthermore, all members of *Ferula* formed two lineages, one lineage contained *F. olivacea*, *F. paeoniifolia*, and *F. kingdon-wardii* (BS = 100, PP = 1), and the other lineage contained all other *Ferula* species (Fig. 7). Moreover, the phylogenetic relationships among non-*Ferula* species conducted in our study were consistent with previous research [43].

### Mericarp morphology

**Fig. 8:**
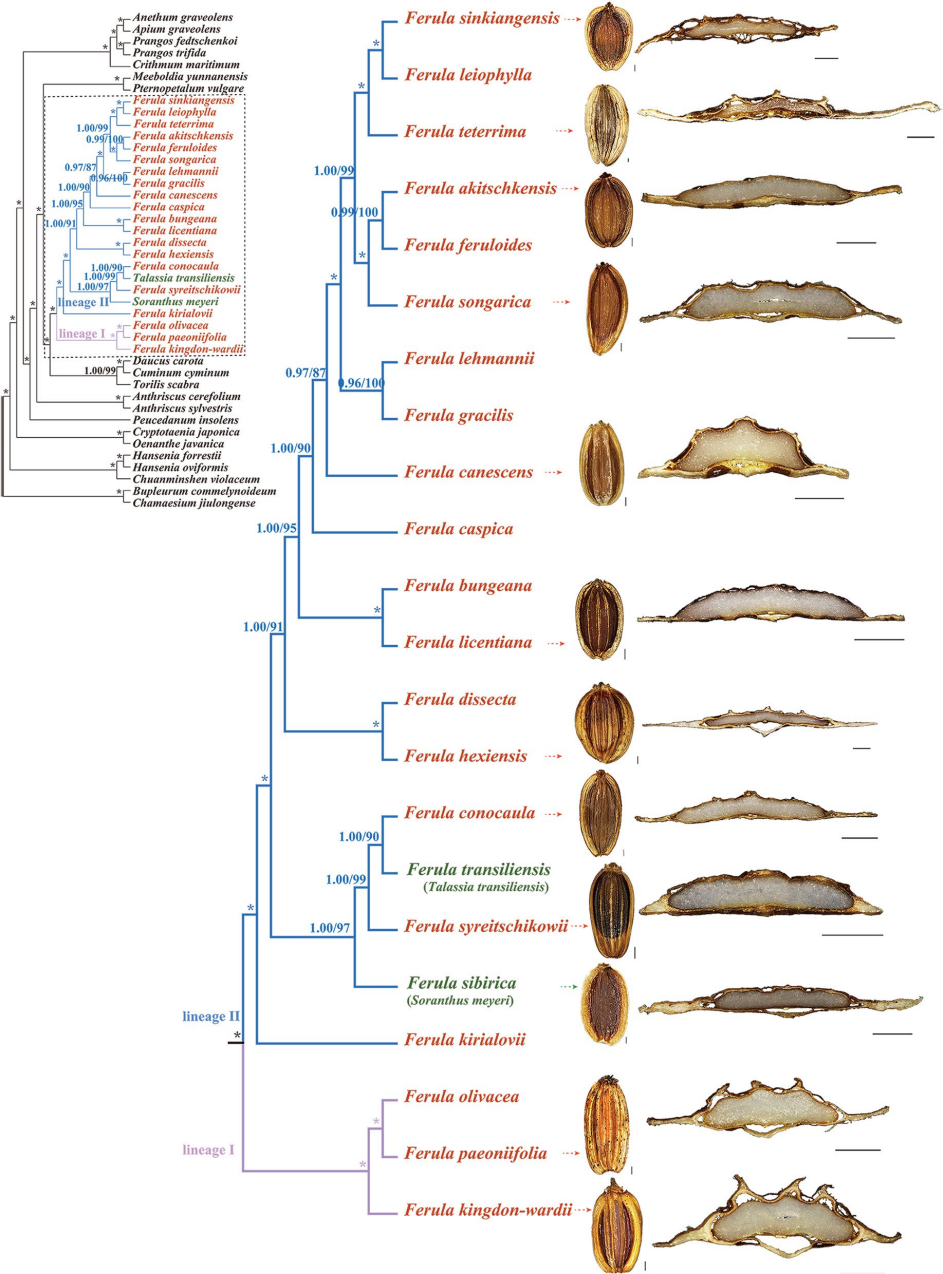
Combination of mericarps and partial plastome CDSs phylogenetic tree from twenty-two species in the black box, with arrows indicating correspondence. Scale bars: dorsal side views = 1 mm, transverse sections = 1 mm

Twenty-two species had mericarps elliptic or ovate, strongly dorsally compressed, and endosperm commissural face plane or slightly concave. Mericarps had a shorter distance between dorsal and median ribs than that of median and lateral ribs. Dorsal and median ribs filiform or sometimes prominent, lateral ribs narrowly or broadly winged. A number of vittae in each furrow (1–4) and commissure (2–12) (Fig. 8, Fig. S2, Table S6).

### Positive selection analysis

**Fig. 9:**
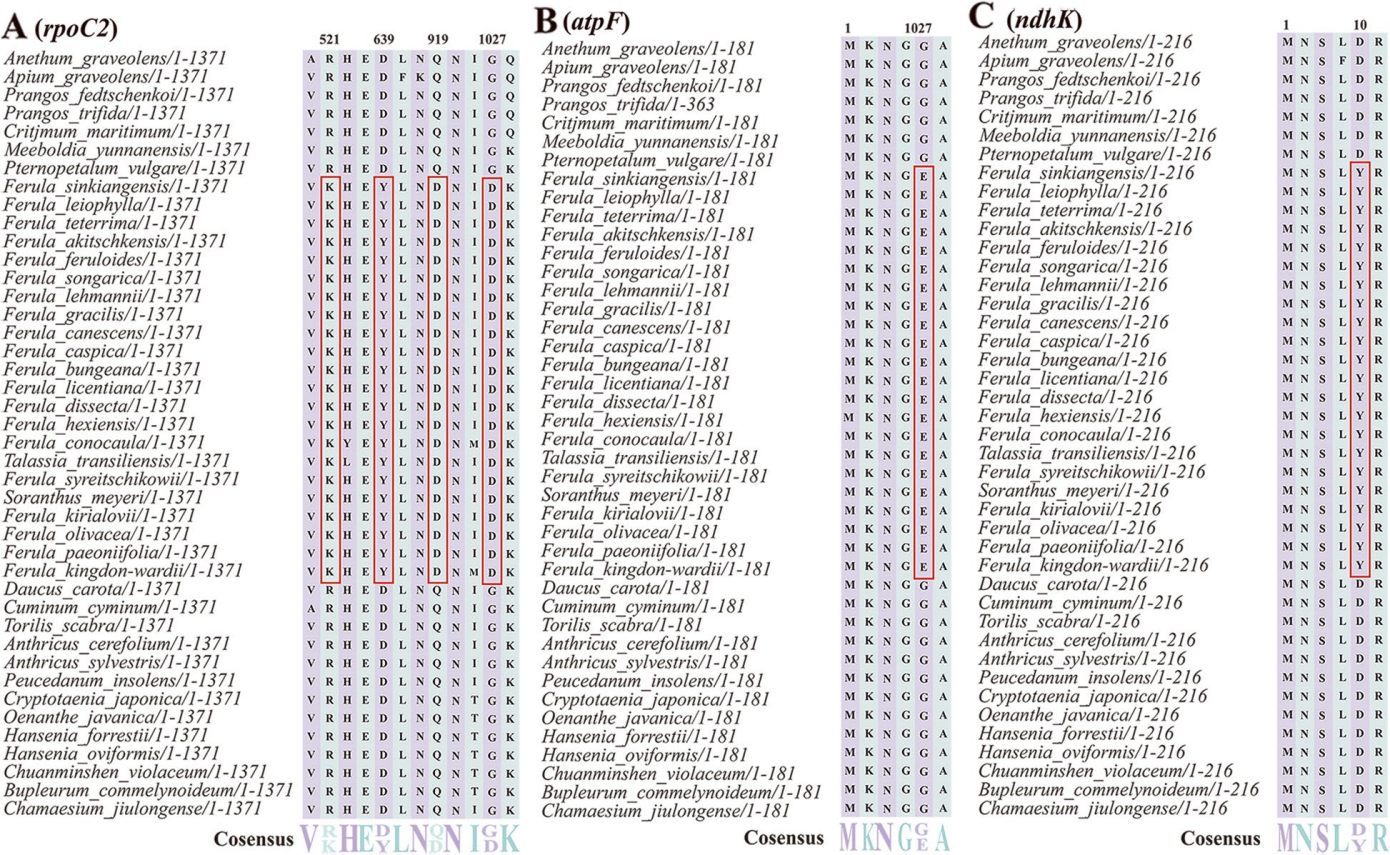
Partial alignment of three out of twelve positively selected genes. **A** Partial aligned amino acids sequences of the *rpoC2* gene; **B** partial aligned amino acids sequences of the *atpF* gene; **C** partial aligned amino acids sequences of the *ndhK* gene. The red blocks indicate the amino acids in *Ferula*, *Talassia*, and *Soranthus* with a high BEB posterior probability

Fifty-two single-copy CDs genes were eventually selected for positive selection analysis. The results showed that the 12 genes (*atpB*, *atpF*, *ndhA*, *ndhC*, *ndhI*, *ndhJ*, *ndhK*, *psbK*, *rpl20*, *rpoB*, *rpoC1*, and *rpoC2*) were observed with significant posterior probabilities suggesting sites positively selected in the BEB test (Table S7). In addition, among these genes, most had one positive selective site, apart from the *rpoC2* that had four positive selective sites (Fig. 9; Fig. S3).

## Discussion

### Comparison of *Ferula* plastomes

In this study, we implemented a comprehensive comparative analysis of 22 plastomes from *Ferula*, *Talassia*, and *Soranthus*. All plastomes possessed a typical circular tetrad structure with two inverted repeat regions, one SSC region, and one LSC region, which is common in other plants [44–46]. Additionally, the gene numbers, type and distribution of large repeats, number and type of SSRs, and codon usage were rather similar among these plastomes. This circumstance is common across other genera in the family Apiaceae [47, 48]. Therefore, these results demonstrated that the plastomes were highly conserved in terms of structure, gene number, type and distribution of large repeat, number and type of SSRs, and codon usage in *Ferula*, *Talassia* and *Soranthus*.

However, we noticed the obvious divergence within the size of 22 plastomes, varying from 160,901 bp (*F. conocaula*) to 167,208 bp (*F. olivacea*). Previous studies inferred that the variation of plastome size was mainly influenced by the following three factors. First, the contraction and expansion of IR regions were the most common reason for the variation of plastome size. For example, a significant expansion was detected in *Pelargonium hortorum*, which resulted in the plastome size increasing [49]. Second, gene losses could lead to the shrinkage of plastome size, especially within several parasitic plants [50]. Third, the indels had an important influence on the plastid genome size within some genera [51, 52]. In this study, the borders of IR/SC regions were slightly varied and gene content was highly conserved, while about 3,020 bp, 2,837 bp, and 2,190 bp deletions in *F. kingdon-wardii*, *F. conocaula*, and *F. caspica* were detected in *ycf15*/ *trn*V, which resulted in the plastome length of the three species being shorter than the other species. As a result, the deletions may be largely responsible for the variation of plastome size in the 22 plastomes.

The SSRs are used to be the molecular markers, in particular, in studies of biogeography and plant population genetics and the identification of species because they have high polymorphic rates [45]. Therefore, those fragments, such as (AAAT)3 only found in the intergenic region (*trn*S /*psbZ*) in *F. kirialovii* and (A)15 observed in the *ndhF* gene only in *T. transiliensis*, may be useful for selecting as molecular markers to differentiate between *Ferula* species in the future.

### Promising DNA barcodes

Accurate species identification has usually been difficult for taxonomists, which was large due to restrictions on incomplete specimens and limitations of field observation of the whole plant. The developing DNA barcoding technology, discriminating species by the short DNA fragments with variable sites [53], looks forward to working out this difficulty. In animals, the mitochondrial gene cytochrome oxidase 1 has been confirmed to be reliable and valid as the DNA barcode for species identification [54, 55]. In plants, the common DNA barcodes including *trn*H-*psbA*, *matK*, and *rbcL* are insufficient to accurately identify species [56, 57]. The variation of the *rbcL* gene was relatively low (Pi = 0.00161) in the 22 studied plant species. As a result, this region may be restricted to accurately delimitating *Ferula* species.

According to the sequence variation, we chose five protein-coding regions (*ycf1*, *ndhF*, *matK*, *rps11*, and *rpl22*) and eight non-coding regions (*ycf15*/*trn*V, *trn*H /*psbA*, *trn*G/*trn*R, *trn*R /*atpA*, *psbI*/*trn*S, *rps15*/*ycf1*, *rps2*/*rpoC2*, and *ycf3*/*trn*S) as the potential identifiers for species in *Ferula*. Among them, the *trn*H-*psbA* region is a member of universal DNA barcodes [57]; *ycf1* and *rpl22*, have been selected as the coming DNA barcodes in some plants [58, 59]. We will examine if these sequences could serve as valid DNA barcodes for species identification in the *Ferula* genus in future research.

### Phylogenetic analyses

Same to previous results obtained by Kurzyna-Młynik et al. [6] based on nrITS data and by Panahi et al. [18] based on nrITS and three plastid DNA *rps16* and *rpoC1* intron, and *rpoB*-*trn*C intergenic spacer, our phylogeny based on plastome data robustly supported that *T. transiliensis* and *S. meyeri* nested in *Ferula* genus. This relationship also showed in our ITS-based phylogenetic tree, although the support of which was weak. Hence, transferring *T. transiliensis* and *S. meyeri* into the *Ferula* genus should be reasonable. And their name should be the *F. transiliensis* [60] and *F. sibirica* [11]. Additionally, our phylogenetic result with high resolution indicated that *T. transiliensis and S. meyeri* were more closely related to *F. conocaula* and *F. syreitschikowii* than the other *Ferula* species. However, due to the limited samples of *Ferula* acquired in our study and maternal inheritance of plastome, their phylogenetic positions within *Ferula* genus need to completely exploit in future studies.

The infrageneric taxonomy of *Ferula* was inconsistent in previous studies. Korovin et al. [19, 61] divided *Ferula* into six subgenera and eight sections based on vegetative features and habits. In The Flora of Reipublicae Popularis Sinica [15], the *Ferula* species grown in China were placed in four subgenera and four sections [15, 19]. However, Panahi et al. [17] proposed a new classification that included four subgenera and eight sections based on molecular phylogenetic results.

In our study, the 22 species were strongly divided into two lineages: one encompassed *F. olivacea*, *F. paeoniifolia*, and *F. kingdon-wardii* (lineage I); the other had the remaining species (lineage II). This result was further supported by species’ geographical distributions and mericarp structures. The members of lineage I are distributed in the alpine meadows and rock cranny of cliffs in Yunnan and Sichuan Provinces [1, 62]; the mericarps of these three species have very prominent dorsal and lateral ribs, and two vascular bundles were present in the dorsal and lateral ribs [63]. Whereas the members of lineage II are located in the gravelly slopes and desert gravels in Xinjiang and other provinces; their mericarps have filiform or slightly prominent dorsal and lateral ribs with one vascular bundle [15, 63]. Combining the robust phylogenetic framework and morphological characteristics, our result strongly supported the establishment of subgenera *Sinoferula* and subgenera *Narthex* [17]. But our result showed that the *F. licentiana* should be placed in the subgenera *Narthex*, and *F. peaoniifolia* should be added into subgenera *Sinoferula*. In addition, our result inferred that the infrageneric taxonomy of *Ferula* genus in Flora of Reipublicae Popularis Sinica [15] was inappropriate.

### The adaptation evolution of *Ferula *plastome

*Ferula* species mostly grow in high-temperature, strong-bright, and drought environments, and thus we speculated several genes were probably under a special evolutionary process [1]. As we expected, 12 genes with significant posterior probabilities for codon sites were identified by the BEB test in our study. Researchers proposed that codon sites with higher posterior probabilities could be considered as positively selected sites, and genes in possession of positively selected sites may evolve under various selection pressure [64]. Therefore, 12 genes detected in our study may have undergone positive selection pressures. The 12 genes comprised two ATP subunit genes (*atpB* and *atpF*), five NADH dehydrogenase genes (*ndhA*, *ndhC*, *ndhI*, *ndhJ*, and *ndhK*), one gene (*psbK*) associated with photosystem II, one gene (*rpl20*) about large subunit of ribosome, and three RNA polymerase subunits genes (*rpoB*, *rpoC1*, and *rpoC2*). Among them, the largest proportion of genes (*ndhA*, *ndhC*, *ndhI*, *ndhJ*, and *ndhK*) are related to the NADH-dehydrogenase subunits. NADH-dehydrogenase subunits were fundamental to the electron transport chain for the generation of ATP, and photosynthesis of plants [65, 66]. Wang et al. [67] found that NADH could induce the PSI cycle electron to divert the electrons to avoid plants being injured and provide the ∆pH for CO2 assimilation for a certain period of time under high-temperature stress. Therefore, these genes under positive selection helped *Ferula* species refrain from injury and thrive in drought and intense light environments. Additionally, several codon sites with significant posterior probabilities were found in *rpo* genes (*rpoB*, *rpoC1*, and *rpoC2*). The *rpoB* gene encodes the β-subunit of RNA Polymerase in plastomes [68], and the *rpoC2* gene encodes another subunit of RNA Polymerase which is responsible for the expression of photosynthetic genes [69]. The previous research indicated that RNA polymerase could not only keep the essential metabolic process to survive, but also regulate the process of gene transcription and expression, for facilitating species to respond to the changing environment conditions [70, 71]. Moreover, via implementing comparative experiments, Gao et al. [72] revealed that the *rpoC2* gene underwent strong positive selection in the sun-loving rice species, and this phenomenon inferred that this gene was important for sun-loving rice species to adapt to the sunlight habitat. Hence, those *rpo* genes under positive selection in our analysis may contribute to adapting the bright environments for *Ferula* species. Furthermore, the *atpF* gene, encoding one of the subunits of H^+^-ATP synthase, played the crucial role in electron transportation, and photorespiration for plants [73]. In a previous study, this gene was positively selected in two evergreen *Quercus* species comparing with two deciduous *Quercus* species, which could help the evergreen species to resist the stress from cold and drought [74]. Generally, the *Ferula* species grow and develop in early spring and live in the arid desert areas [15, 75], thereby the *atpF* gene may be significant in environment adaptation of *Ferula* species. In brief, these positively selected genes have been beneficial to the development and reproduction of *Ferula* species, and played an important role in adapting to the harsh environment where *Ferula* species grow.

## Conclusion

In our study, we sequenced and assembled 22 plastomes of *Ferula*, *Talassia*, and *Soranthus* species. Based on the comparative analysis of plastomes, we observed conservation in genome structure, gene number, codon usage, and repeats types and distribution, but variation in plastomes size, GC content, and the SC/IR boundaries. Thirteen mutation hotspot regions were detected and has potential as DNA barcodes for species identification in *Ferula* and related genera. Based on the phylogenetic analysis for *Ferula* using 22 plastomes and 62 ITS sequences, we agreed with some previous studies that *Talassia* and *Soranthus* should be placed into *Ferula*. Our result also supported the monophyly of subgenera *Sinoferula* and subgenera *Narthex*. The phylogeny reconstructed by the plastomes highlighted the strength of the plastome that possessed the more variable sites and greatly resolved the phylogeny of studied species. In addition, twelve genes with significant posterior probabilities for codon sites helped *Ferula* species to adapt to their harsh environments. Our study offers a new perspective for further study in phylogeny and evolution of *Ferula* species.

## Methods

### Plant materials and DNA extraction

Fresh leaves from adult plants of the 22 species were collected from each yield site. Then, the leaves were immediately dried using silica gel for DNA extracting. The total genomic DNA was extracted from the dried leaf tissue using a plant DNA extraction kit (Cwbio Biosciences, Beijing, China). The formal identification of those samples collected was undertaken by Associate Professor Songdong Zhou (Sichuan University). The Voucher specimens were deposited at the herbarium of Sichuan University (Chengdu, China), and their deposition numbers were listed in the Additional file 11: Table S8. The newly sequenced 22 ITS have been submitted to NCBI (Additional file 8: Table S5).

### Plastome genome sequencing and assembling

The raw reads of 22 newly sequenced species were generated from the Illumina HiSeq X Ten platform (paired-end, 150 bp) at Novogene (Tianjin, China). The raw reads were filtered using fastP version v0.15.0 (-n 10 and -q 15) to yield clean reads [76]. Then clean reads were used to assemble plastomes using NOVOPlasty v2.6.2 [77] with default parameters and the *rbcL* gene (MK749921.1) of *F. bungeana* downloaded from NCBI as seed. The assembled genomes were initially annotated by the PGA [78], and then adjusted manually in Geneious v9.0.2 [79]. Using the same method, the plastomes of non-*Ferula* obtained from the NCBI were re-annotated. Finally, the plastid genome maps were drawn using Chloroplot [80].

### Repeat sequences and codon usage

The Perl script MISA (http://pgrc.ipk-gatersleben.de/misa/) was used to analyze simple sequence repeats (SSRs) in the plastome sequences. The parameters of SSRs were set as follows: 10, 5, 4, 3, 3, and 3, in response to mono-, di-, tri-, tetra-, penta-, and hexanucleotides, respectively. The REPuter online program [81] was used to search repeat sequences including (F) forward, (P) palindromic, (R) reverse, and (C) complementary repeats. The parameters were as follows: (1) a repeat size of over 30 bp; (2) two repeats with more than 90% sequence identity; and (3) Hamming distance = 3. Then, the protein-coding genes were extracted from the 22 plastid genomes for codon analysis by the CodonW v1.4.2 program [82].

### Genome structure and sequence diversity

The IR region contraction and expansion at the border of the plastome were analyzed by the online program IR scope [83]. The size and position of the gene were then manually adjusted. The sequence identity of whole plastomes was detected and visualized by the online program m-VISTA [84] in Shuffle-LAGAN mode, with the *F. sinkiangensis* as a reference. Nucleotide diversities of the coding genes and intergenic regions were calculated by DnaSP v5 [85].

### Phylogenetic analysis

To investigate the phylogeny of *Ferula*, 42 plastomes and 62 nuclear ITS sequences were used to reconstruct the phylogenetic tree (Table S5). *Chamaesium jiulongense* X. L. Guo & X. J. He, *Bupleurum commelynoideum* de Boiss. were selected as the outgroups to root the phylogenetic tree according to the results of Zhou et al. [86]. For plastome data, 80 single-copy protein-coding sequences (CDs) commonly shared by the 42 plastomes were extracted using Phylosuite v.1.2.2 [87] and then respectively aligned by MAFFT v7.221 [88]. These alignments were concatenated as a super matrix by Phylosuite v.1.2.2 [87]. The nrITS sequences were aligned by MAFFT v7.221 [88].

The prepared data sets of CDs and nrITS were then subjected to Maximum-Likelihood (ML) analyses and Bayesian Inference (BI). For ML analysis, the phylogenetic trees were generated by RAxML 8.2.8 [89] with the GTRGAMMA model, as suggested in the RAxML manual, and 1,000 bootstrap replicates. The BI analysis was conducted using MrBayes v.3.2.5 [90], with the TVM + I + G and GTR + I + 0 substitution models determined by Modeltest v3.7 [91] for plastomes and ITS, respectively. Markov chain Monte Carlo (MCMC) algorithm was run for one million generations, with one tree sampled every 100 generations. The first 25% of trees were discarded as burn-in, and the remaining trees were used to build the consensus tree. The phylogenetic tree was displayed and edited in FigTree v1.4.2 [92].

### Positive selected analysis

The Optimized Branch-Site model [93] and the Bayesian Empirical Bayes (BEB) [64] method were used to identify genes that were positively selected in *Ferula* species compared to the non-*Ferula* species. Single-copy protein-coding regions of 42 plastomes were extracted and then aligned using the ClustalW [94] with the amino acid codons. Then the alignments of sequences were trimmed. Finally, the trimmed alignments were used to implement the positive selection analysis by the CODEML algorithm in the PAML package [95] in EasyCodeml [96] with the branch-site model and the *Ferula* clade designed as the foreground branch. The BEB method was used to compute the posterior probabilities of amino acid sites to confirm whether these sites were selected positively and with high posterior probabilities [64]. The likelihood-ratio tests (LRT) were implemented based on Lan et al. [97], as a result, if the gene was with a *p*-value < 0.5, it would be certified as the positively selected gene. We then used Jalview v.2.11.1.7 [98] to view the amino acid sequences of positively selected genes.

### Morphological observations of mericarps

The whole structures of dorsal and commissural side views, and anatomical structures including transverse section, rib shape, and vittae of mericarps in 12 species were observed and photographed via a stereomicroscope (SMZ25, Nikon Corp., Tokyo, Japan). These mature mericarps were selected randomly and measured by the KaryoType [99]. Mericarp terminology is based on Kljuykov et al. [100].

## Supplementary Information

**Additional file 1:** **Fig. S1.** Phylogenetic tree reconstruction of the 62 taxa inferred from Bayesian inference (BI) analyses and Maximum likelihood (ML) based on nuclear internaltranscribed spacer (ITS) sequences. Numbers indicate Bayesian posterior probabilities (PP) and mamxium likelihood bootstrap values (BS), and (*) indicates maximumsupport in both two analysis, and (-) indicates mamxium likelihood bootstrap values (BS) less than 50 in Maximum likelihood (ML) analyses.

**Additional file 2:** **Fig. S2.** Morphological features of mericarps of twelve species. (A) Dorsal side views ofmericarps. (B) commissural side views of mericarps. (C) transverse sections. Scale bars:A=1.0 mm;B=1.0 mm; C=0.5 mm.

**Additional file 3:** **Fig. S3.** Partial alignment of amino acids sequences in another nine positively selected genes. (A-J): *atpB*, *ndhA*, *ndhC*, *ndhI*, *ndhJ*, and *psbK*, *rpl20*, *rpoB*, and *rpoC1*. The red blocks indicate the amino acids in twenty-two species with a high BEB posterior probability. 

**Additional file 4:** **Table S1.** The list of gene content in the twenty-two plastomes.  

**Additional file 5:** **Table S2.** Simple sequence repeats (SSRs) distribution in the twenty-two plastomes. 

**Additional file 6:** **Table S3.** The distribution of repeat sequences in the 22 plastomes.

**Additional file 7:** **Table S4.** Codon usage and relative synonymous codon usage (RSCU) values of protein-coding genes of the 22 plastomes. 

**Additional file 8:** **Table S5. **The nrITS Genbank accession numbers of all species used in phylogenetic analysis. 

**Additional file 9:** **Table S6.** Synopsis of the morphological information from the 22 species. 

**Additional file 10:** **Table S7.** The result of  positive selection analysis based on the branch-site model. 

**Additional file 11:** **Table S8.** Information for sample collections and specimen vouchers of the 22 species. 

## Data Availability

Twenty-two annotated plastomes have been submitted to NCBI (https://www.ncbi.nlm.nih.gov) with accession numbers: OP324722-OP324743; newly sequenced 22 nrITS have been submitted to NCBI (https://www.ncbi.nlm.nih.gov) with accession numbers: OP341492-OP341513 (Additional file 8: Table S5).

## Abbreviations

BEB: Bayes empirical bayes
BI: Bayesian inference
bp: Base pair
BS: Branch support
CDS: Protein-coding sequences
IR: Inverted repeat
ITS: Internal transcribed spacer
LRT: Likelihood ratio test
LSC: Large single copy
MCMC: Markov chain Monte Carlo
ML: Maximum Likelihood
Pi: Nucleotide diversity
PP: Posterior probability
rRNA: Ribosomal RNA
RSCU: Relative synonymous codon usage
SSC: Small single copy
SSR: Simple sequence repeat
tRNA: Transfer RNA
