# Supplementary material for: The plastid genome of twenty-two species from Ferula, Talassia, and Soranthus: comparative analysis, phylogenetic implications, and adaptive evolution
Source: BMC Plant Biol. 2023 Jan 5;23:9. doi: 10.1186/s12870-022-04027-4 (PMC9814190; doi:10.1186/s12870-022-04027-4)
Supplement: Supplementary file 1 — Additional file 1: Fig. S1. Phylogenetic tree reconstruction of the 62 taxa inferred from Bayesian inference (BI) analyses and Maximum likelihood (ML) based on nuclear internaltranscribed spacer (ITS) sequences. Numbers indicate Bayesian posterior probabilities (PP) and mamxium likelihood bootstrap values (BS), and (*) indicates maximumsupport in both two analysis, and (-) indicates mamxium likelihood bootstrap values (BS) less than 50 in Maximum likelihood (ML) analyses. [file 12870_2022_4027_MOESM1_ESM.pdf]

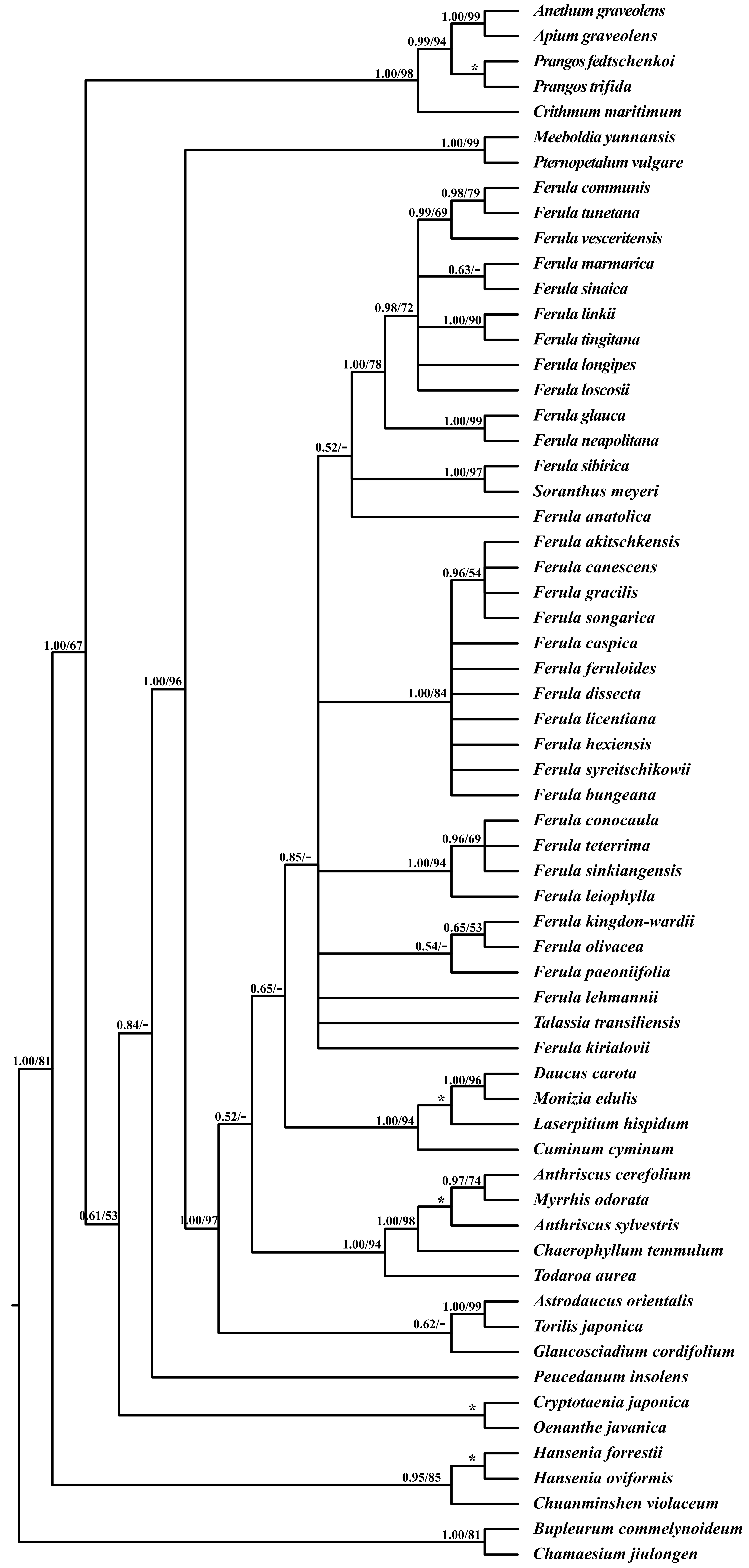


**Fig. S1** Phylogenetic tree reconstruction of the 62 taxa inferred from Bayesian inference (BI) analyses and Maximum likelihood (ML) based on nuclear internal transcribed spacer (ITS) sequences. Numbers indicate Bayesian posterior probabilities (PP) and mamxium likelihood bootstrap values (BS), and (*) indicates maximum support in both two analysis, and (-) indicates mamxium likelihood bootstrap values (BS) less than 50 in Maximum likelihood (ML) analyses.
