# Supplementary material for: The plastid genome of twenty-two species from Ferula, Talassia, and Soranthus: comparative analysis, phylogenetic implications, and adaptive evolution
Source: BMC Plant Biol. 2023 Jan 5;23:9. doi: 10.1186/s12870-022-04027-4 (PMC9814190; doi:10.1186/s12870-022-04027-4)
Supplement: Supplementary file 2 — Additional file 2: Fig. S2. Morphological features of mericarps of twelve species. (A) Dorsal side views ofmericarps. (B) commissural side views of mericarps. (C) transverse sections. Scale bars:A=1.0 mm;B=1.0 mm; C=0.5 mm. [file 12870_2022_4027_MOESM2_ESM.pdf]

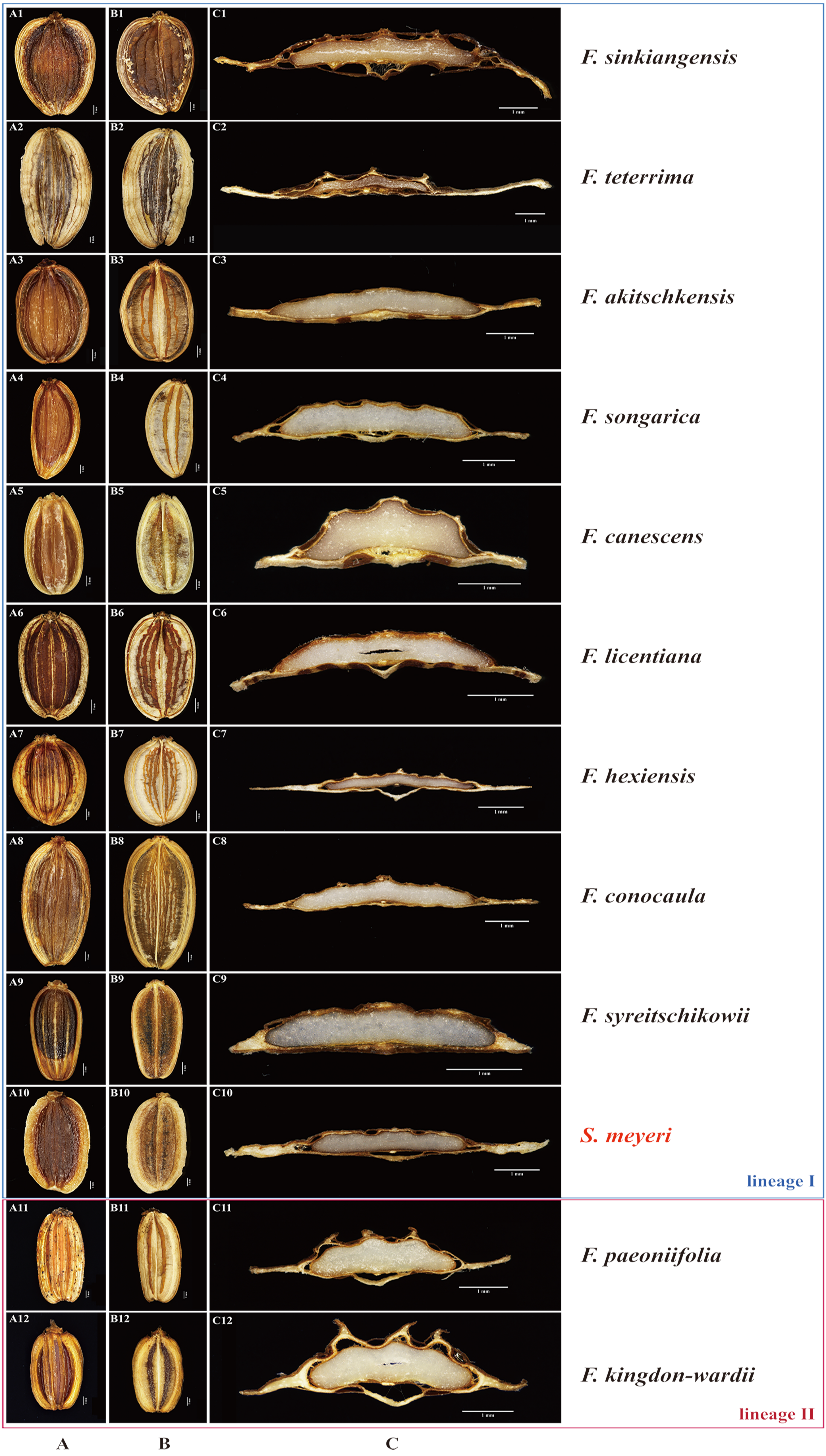

Fig. S2 Morphological features of mericarps of twelve species. (A) Dorsal side views of mericarps. (B) commissural side views of mericarps. (C) transverse sections. Scale bars: A=1.0 mm;B=1.0 mm; C=0.5 mm
