# Supplementary material for: The plastid genome of twenty-two species from Ferula, Talassia, and Soranthus: comparative analysis, phylogenetic implications, and adaptive evolution
Source: BMC Plant Biol. 2023 Jan 5;23:9. doi: 10.1186/s12870-022-04027-4 (PMC9814190; doi:10.1186/s12870-022-04027-4)
Supplement: Supplementary file 3 — Additional file 3: Fig. S3. Partial alignment of amino acids sequences in another nine positively selected genes. (A-J): atpB, ndhA, ndhC, ndhI, ndhJ, and psbK, rpl20, rpoB, and rpoC1. The red blocks indicate the amino acids in twenty-two species with a high BEB posterior probability. [file 12870_2022_4027_MOESM3_ESM.pdf]

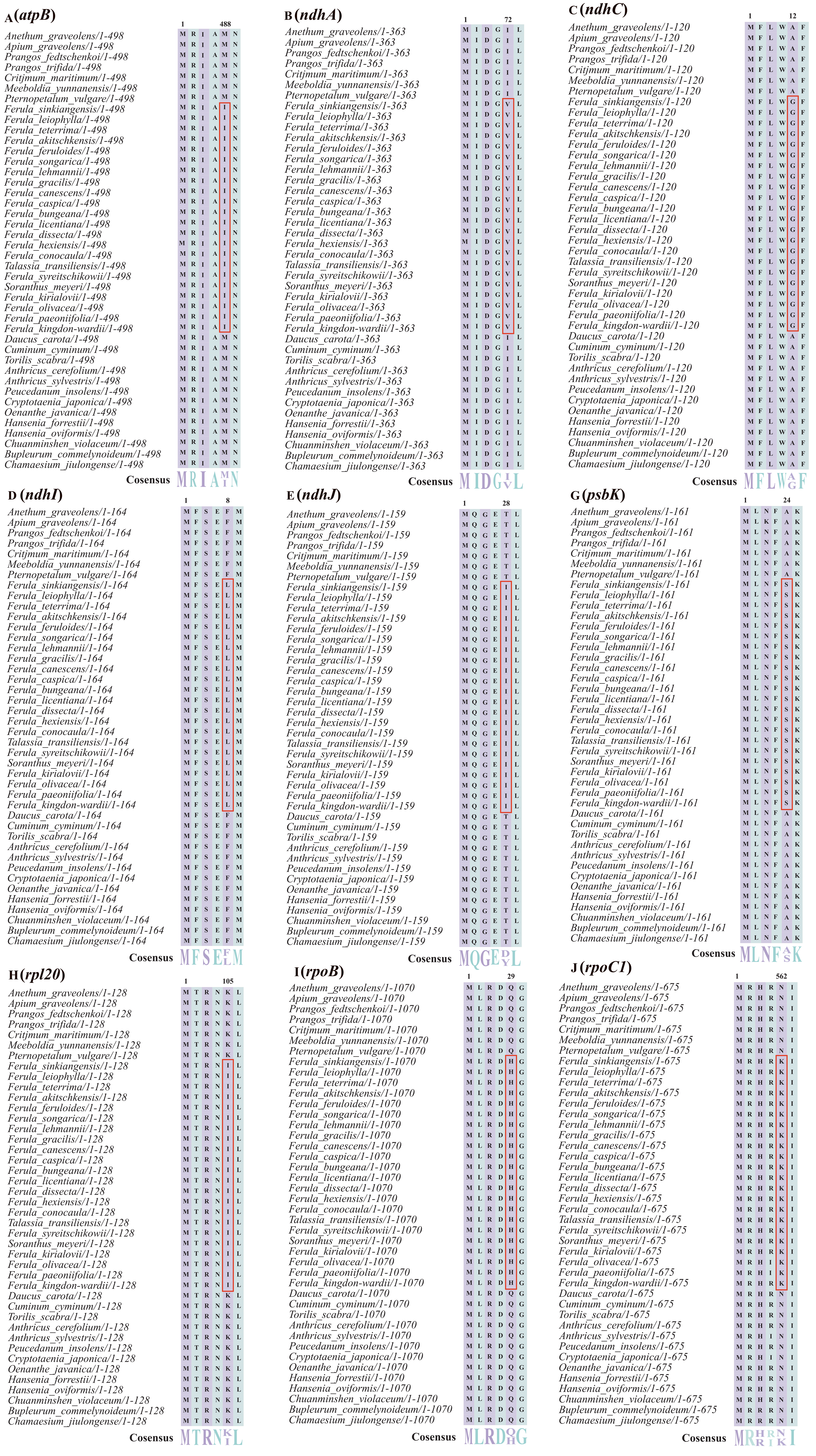

Fig. S3 Partial alignment of amino acids sequences in another nine positively selected genes. (A-J): *atpB*, *ndhA*, *ndhC*, *ndhI*, *ndhJ*, and *psbK*, *rpl20*, *rpoB*, and *rpoC1*. The red blocks indicate the amino acids in twenty-two species with a high BEB posterior probability.
